# Analytic and Holistic Thinkers: Differences in the Dynamics of Heart Rate Complexity When Solving a Cognitive Task in Field-Dependent and Field-Independent Conditions

**DOI:** 10.3389/fpsyg.2021.762225

**Published:** 2021-11-26

**Authors:** Anastasiia V. Bakhchina, Vladimir V. Apanovich, Karina R. Arutyunova, Yuri I. Alexandrov

**Affiliations:** ^1^Laboratory of Neural Bases of Mind Named After V.B. Shvyrkov, Institute of Psychology of Russian Academy of Sciences, Moscow, Russia; ^2^Department of Psychophysiology, National Research University Nizhny Novgorod State University Named After N.I. Lobachevsky, Nizhny Novgorod, Russia; ^3^International Laboratory of Social Neurobiology, Institute of Cognitive Neuroscience, National Research University Higher School of Economics, Moscow, Russia

**Keywords:** analytic thinking, holistic thinking, visual discrimination, reaction time, heart rate variability, complexity, entropy analyses, system-evolutionary theory

## Abstract

Analytic and holistic thinking styles are known to be associated with individual differences in various aspects of behavior and brain activity. In this study, we tested a hypothesis that differences in thinking styles may also be manifested at the level of neuro-visceral coordination. Heart rate variability (HRV) was compared between analytic and holistic thinkers at rest, during a simple motor choice reaction time task and when solving cognitive choice reaction time tasks in conditions with varying instructions contrasting the role of the field when evaluating objects. Participants (*N* = 52) with analytic and holistic thinking styles were equally successful at solving the cognitive tasks but response times were longer in the analytic group, compared to the holistic group. Heart rate complexity, as measured by sample entropy, was higher in the analytic group during the cognitive tasks but did not differ from the holistic group at rest or during the simple motor task. Analytic participants had longer response times and higher heart rate complexity when evaluating objects in relation to the field than when evaluating objects irrespective to the field. No difference in response times or heart rate complexity between tasks was observed in the holistic group. Our findings demonstrate that differences in individual behavior, including those related to holistic and analytic thinking styles, can be reflected not only in brain activity, as shown previously using fMRI and EEG methods, but also at the level of neuro-visceral coordination, as manifested in heart rate complexity.

## Introduction

A theoretical model of analytic vs. holistic thinking was proposed by Nisbett et al. (2001) to explain cognitive differences observed between individuals from Eastern and Western cultures. Some key elements that distinguish analytic and holistic thinkers include their attention to relationships between objects and the field these objects belong to, understanding of causality, perception of changes and explanation of contradictions. In general, analytic thinkers tend to focus their attention on an object rather than the field it belongs to; explain causal relationships primarily via internal dispositions of an actor; perceive most objects as independent, often observing linear changes in them; and rely on formal logic approach to resolve contradictions by choosing one alternative over another. In contrast, holistic thinkers tend to pay more attention to the relationships between an object and the field; consider a greater amount of information and complex relationships between an actor and the surrounding situation when determining causality; perceive cyclical changes of elements and their interconnections with one another; and try to reconcile contradictions by looking for compromises when contradictory opposites exist. Although initially applied in cross-cultural studies, this model was also shown to differentiate thinking styles between individuals within cultures (e.g., Choi et al., 2007).

Differences in cognitive performance between holistic and analytic individuals have been demonstrated across various tasks involving attention (Ji et al., 2000; Masuda and Nisbett, 2001), categorization (Choi et al., 1997; Ji et al., 2001; Norenzayan et al., 2002), causal attribution (Norenzayan et al., 2002), tolerance of contradiction (Peng and Nisbett, 1999), etc. These differences are reflected in problem solving strategies and brain activity of analytic and holistic thinkers. EEG and fMRI studies reported differences in brain activity related to thinking styles during cognitive tasks (Apanovich et al., 2021) and in socio-emotional contexts (Baranski and Petrusic, 1999; Haun et al., 2006; Winawer et al., 2007; Tan et al., 2008; Apanovich et al., 2018; Huang et al., 2019; Hsieh et al., 2020). Psychophysiological processes that occur outside the brain are not always considered in cognitive studies but there is substantial evidence demonstrating that neuro-visceral coordination plays an important role in the organization and regulation of individual behavior (Bakhchina et al., 2018; Forte et al., 2019). We have come across only one study reporting results on visceral dynamics observed in analytic and holistic individuals, which included analyses of respiration and heart rate (Bacha-Trams et al., 2018). In the current work, we aim to compare heart rate variability (HRV) and complexity between holistic and analytic individuals to test whether differences in cognitive performance and brain activity shown in many previous studies are manifested in neuro-visceral processes. On the one hand, it will provide an opportunity to consider physiological bases of analytic and holistic thinking wider shedding light on visceral peculiarities of individuals with different cognitive styles. On the other hand, it will assist in the development of the embodiment theory that views cognition as a whole organism process with the body playing an important role in decision-making and other cognitive activities (Macedonia, 2019).

Heart rate variability (HRV) reflects the dynamics in the time intervals between adjacent heartbeats that are in coherence with the functioning of the entire body. It is widely accepted that the source of HRV originates in the regulation of transport of resources through the body in order to adapt an organism’s functioning to external challenges by achieving optimal performance ratio. From the physiological perspective, the primary origin of HRV is related to the activity of the autonomic nervous system through its sympathetic and parasympathetic parts (Thayer and Lane, 2009). Various cortical areas in the brain contribute to the regulation of heart rate (e.g., see review in Smith et al., 2017) and changes in HRV indexes during cognitive and socioemotional tasks are shown to correlate with some aspects of task performance (Kemp et al., 2010; Arutyunova et al., 2020; Hilgarter et al., 2021). Therefore, the analysis of HRV has become a popular non-invasive tool in psychophysiological studies (Laborde et al., 2017). In our previous work, addressing the problem of brain-heart interactions from the positions of the system-evolutionary theory, we proposed a model explaining HRV in relation to neuronal processes involved in the orchestration of an organism’s behavior (Bakhchina et al., 2018).

The system-evolutionary theory (Shvyrkov, 1990; Aleksandrov, 2006), building upon the foundations of P.K. Anokhin’s theory of functional systems (Anokhin, 1974), proposes that morphologically different components of the brain and the rest of the body comprise functional systems in order to achieve a positive result, i.e., adaptive organism-environment relations. A functional system is understood as a dynamic organization of activity of neurons and other cells across different anatomical localizations which provides the achievement of an adaptive result for the whole organism. Each novel way of adaptive organism-environment interaction has a potential to underlie the formation of a new functional system in the process of learning. Subsequent actualization of this new functional system underlies execution of the corresponding behavioral act in order to achieve the adaptive interaction with the environment. In this way, HRV originates in the cooperation of the heart with the other components of actualized functional systems, including neuronal groups. Variable changes in heart rate are viewed as a result of heart coordination with changes in the sets of activated neurons distributed across the cerebral cortex and subcortical structures and specialized in relation to particular behavior. Our previous studies (Bakhchina et al., 2018; Arutyunova et al., 2020) demonstrate that, out of existing and widely used HRV indexes, non-linear metrics, such as entropy measures, most accurately reflect changes in behavior and cognitive performance. Other authors also reported that non-linear HRV metrics (e.g., entropy and fractal dimension) are more informative for the studies of behavior than statistical and frequency measures of HRV (Pham et al., 2021). In accordance with the system-evolutionary theory, entropy measures of HRV reflect the system characteristics of individual experience actualized in current behavior. Behavior acquired at earlier stages of development is more automated and intuitively implemented than newly formed behavior, and vice versa behavior acquired at later stages of development is more differentiated and detailed than earlier acquired behavior. Values of heart rate complexity observed during newer behavior are expected to be higher (Bakhchina et al., 2018; Arutyunova et al., 2020). Thus, comparative analyses of HRV entropy measures can be used to reconstruct the relative level of differentiation of the individual experience actualized during problem-solving. Reflecting the complexity of neuro-visceral coordination, heart rate entropy allows us to test the hypotheses about the systems orchestration of behavior in individuals with analytic and holistic thinking.

In this work, we compared heart rate complexity between groups of participants with holistic and analytic thinking styles during performance of holistic (field-dependent) and analytic (field-independent) tasks. Based on the assumption that functional systems are comprised by different components of the brain and the rest of the body, our hypothesis was that cognitive and behavioral specificity of analytic and holistic thinking styles is not only manifested in brain activity but reflects the system organization of individual experience at the level of the whole organism. More specifically, we hypothesized that there are differences in heart rate complexity between analytic and holistic thinkers when solving cognitive tasks and these differences are related to their task performance. We expected analytic individuals to be faster and more successful at solving tasks in a field-independent condition and holistic individuals to be faster and more successful in a field-dependent condition. We hypothesized that the cognitive effort and the system complexity of current behavior would be manifested in neuro-visceral activity so that higher HRV complexity would correspond to performance in the tasks that are harder for individuals: field-dependent tasks for analytic thinkers and field-independent tasks for holistic thinkers.

## Materials and Methods

### Ethics Statement

All of the participants gave written informed consent to take part in the study after receiving an explanation of the procedures.

The study was conducted in accordance with the Declaration of Helsinki. The Ethics Committee of Institute of Psychology of Russian Academy of Sciences (Moscow) approved the experimental protocols and the specific consent procedure used in this study and assessed it as safe for the participants’ psychic and physical health. All of the participants were paid for their participation (500 rubles).

### Participants

We studied 52 healthy participants (25 women; 18–34 years old: *M* = 21.2, *SD* = 4.34). All participants were Russian citizens speaking Russian as their native language. None of them reported any history of neurological or psychiatric disorders. All participants had either normal or corrected-to-normal vision. One participant was excluded from the analyses due to corrupted ECG recordings. The holistic and analytic groups were formed on the basis of participants’ answers to the items of AHS questionnaire (see below). It was not possible to calculate AHS scores for 5 of the participants because they used the option “difficult to answer” too often. Finally, the electrophysiological data of 46 participants were included into the analyses.

### Experimental Procedure

**FIGURE 1:**
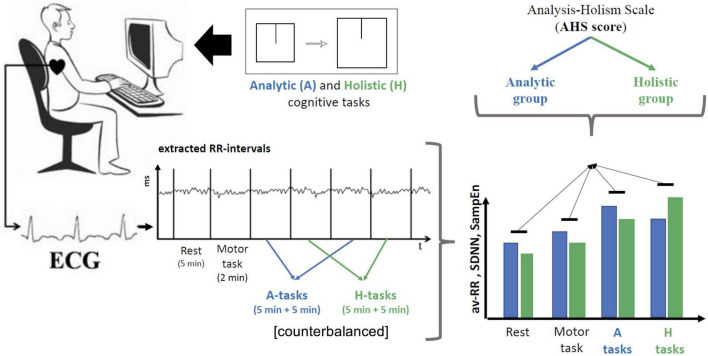
Experimental design and procedure. Prior to the experiment, participants (*N* = 52) filled an Analysis-Holism Scale (AHS) questionnaire and were divided into two groups: those with scores higher than median for the sample comprised the holistic group and the rest comprised the analytic group. ECG was recorded at rest (5 min, while sitting down with closed eyes without moving), during a simple motor task (2 min), and in the process of solving four blocks of cognitive tasks (5 min per each block). Cognitive tasks included presentation of two square frames (fields) containing lines (objects), sizes of which varied; participants were asked to compare objects either in relation to their fields (H-tasks) or irrespective of the fields (A-tasks). Response times, success rates and the dynamics of HRV indexes (av-RR, SDNN and SampEn) were compared between the two groups of participants.

Experimental design and procedures are illustrated in Figure 1.

ECG was recorded in 6 stages: at rest (5 min, while sitting down with closed eyes without moving), during a simple motor task involving familiarizing with keys to be used in the following experimental sessions (2 min), and in the process of four experimental sessions (5 min per session, two blocks of analytic tasks and two blocks of holistic tasks).

Four experimental sessions included solving tasks in two analytic and two holistic conditions. Analytic tasks were constructed to allow successive stages in the process of searching for a solution; holistic tasks could only be solved in one stage, simultaneously (Apanovich et al., 2020). It is also shown that people with analytic and holistic thinking have different attention focus signatures: the former have higher scores in tasks that require abstraction from the context (i.e., background, or field), whereas the latter are more successful when the background is necessary as a part of attention focus (Kitayama et al., 2003). The same framed-line test principle (Kitayama et al., 2003) had been fruitfully employed previously in psychological and psychophysiological research (Hedden et al., 2008). We selected this type of tasks because it constitutes a simple behavioral model which allows to manipulate the use of the field while evaluating objects as one of the basic features contrasting analytic and holistic thinking.

In all tasks, participants were presented with two frames of different sizes which contained vertical lines. A screenshot example of one of the experimental tasks is presented in Figure 1. The first line was presented 900 ms after the “Attention” signal (a white cross on a dark background) and the second line was presented in a random interval in the range from 700 to 900 ms after the first line. After two successively presented images, a participant was prompted to respond, whether one of the aspects within the two images was similar or different. The response was provided by pressing one of two keys (left or right - counterbalanced between tasks). The criterion for similarity/difference was determined in the instruction: in the analytic condition, participants were asked to compare the absolute length of lines, ignoring the frames, whereas in the holistic condition, participants compared lengths of the lines relative to the frames. Presentation order of analytic and holistic tasks was counterbalanced between participants. Thus, the main factor which induced subjective differences in analytic and holistic tasks between these experimental conditions was the instruction: an object was evaluated either in relation to the field or independently of the field.

After reading the instructions, an experimenter started a training trial and asked the participants whether presented figures were similar or different according to the principle indicated in the instructions (analytic or holistic). The experiment began only after the participant understood the instructions and correctly performed the training task.

Response times (RT) were analyzed for three conditions: simple motor trial, blocks of holistic and analytic tasks. Medians and between-subject variances were also calculated for all of the parameters.

Rest, or baseline, condition was viewed as a situation when an individual was not acquiring a new skill. A simple motor task was used, firstly, to control the response rate with the right and left hands, and, secondly, it was used as a “neutral” condition which did not involve thinking styles. Analytic and holistic tasks are considered as situations of operating and developing thinking styles based on prior individual experience.

### Classification of the Participants Into the Analytic and Holistic Groups

All the participants filled the Analysis-Holism Scale (AHS) questionnaire prior to the experiment.

Choi and colleagues (Choi et al., 2007) developed the AHS questionnaire to measure key differences in individuals’ thinking styles and showed that AHS scores correlated with holistic and analytic patterns of performance in cognitive tasks. They also highlighted that individuals’ thinking styles vary not only across cultures but also within the same cultures. AHS score characterizes individual thinking styles on a continuum between analytic and holistic poles so that some individuals are closer to one of the poles while others score around the middle combining more or less features of both. AHS is based on a four-component model of cognition proposed by Nisbett et al. (2001). The questionnaire had been translated and adapted on a sample of Russian participants in 2014–2017 (Apanovich et al., 2017) and was effectively used in our studies, including analysis of EEG (Apanovich et al., 2016; Apanovich et al., 2018) and tasks performance (Apanovich et al., 2020). In our work, we studied analytic and holistic thinking styles within one culture, but, as mentioned above, the AHS can be used to measure both cross-cultural and within-cultural differences (Choi et al., 2007).

The AHS questionnaire consists of 24 statements. Participants were asked to rank whether they agree or disagree with each of the statements on a 7-step Likert scale (from 1 = “completely disagree” to 7 = “completely agree,” with 4 = “difficult to answer” in the middle). The test provides a total score of analytic-holistic thinking, and four subscores of the individual components of the analytic-holistic thinking type. The detailed description of the subscales can be found in Choi et al. (2007). The highest score for each scale represents a holistic pole, and the lowest–the analytic pole. Participants who responded “difficult to answer” 6 or more times were excluded from subsequent analyses (*N* = 5).

The internal consistency reliability (Cronbach’s alpha coefficient) of the AHS in our study was 0.696.

On the basis of AHS scores (Med = 113, Min = 86, Max = 148, 25% = 106, 75% = 123), the participants were divided into two groups with a cut-off at the median value for all participants so that we had a holistic group and an analytic group, containing 23 participants each.

### ECG Acquisition

ECG was recorded using monopolar surface silver chloride electrode in the medial part of the thorax. The electrode had conductive adhesive hydrogel for better signal conductivity. The sampling frequency ECG was 250 Hz with 0.1--70 Hz band-pass range and 50 Hz notch filter. Encefalan EEGR--19/26 for ECG recording was used.^1^

### Heart Rate Variability Analyses

For HRV analyses, we used SampEn as an index of complexity, SDNN as an index of variation, and averaged RR-intervals as an index of mean frequency of the heart rate. It is accepted that heart rate complexity measured with non-linear algorithms can correlate with statistical measures of HRV, such as SDNN, but these correlations are not linear (Acharya et al., 2006). Such modes of heart activity exist when variability is the same, but complexity is different, and vice versa. Heart rate complexity and variability reflect different aspects of temporal dynamics: for example, SDNN is a statistical measure of HRV which is modulated by breathing, or respiratory arrhythmia, while SampEn is an index of heart rate complexity which is independent of respiratory arrhythmia (Richman and Moorman, 2000). Thus, SampEn and SDNN are both HRV indexes partly complementing each other when describing heart activity in relation to behavior.

ECG processing, RR-intervals detection and HRV calculations were made using open-sourced python library neurokit 2 0.1 (Makowski et al., 2021).

Raw ECG signal data obtained for experimental periods (rest, simple motor task, two blocks of analytic tasks and two blocks of holistic tasks) underwent signal preprocessing. It was detrended to remove the linear trend and mean value from the signal. After detrending, it was filtered using a Butterworth band-pass filter with a gain of 1 and frequency range between 5 and 40 Hz to remove the baseline drift, white noise, and any motion artifacts. In the resulting ECG, R-peak detection was based on the Pan and Tomkins algorithm (Pan and Tompkins, 1985).

Acquired sequences of RR-intervals were pre-processed before proceeding to the analyses with the aim to select sequences that were free from artifacts. Sequences with abnormal beats and any artifacts (ectopic beats, motion artifacts, and coughs) were excluded from the analyses. Sequences containing RR-intervals that did not satisfy the condition (1) were excluded from the analyses.


(1) $$\left|R\,R_{i}-R\,R_{i-1}\right|<0.7*\frac{R\,R_{i}-R\,R_{i-1}}{2},$$

Heart rate complexity was described using sample entropy (SampEn) as a set of measures of system irregularity reporting on similarity in time series. SampEn can be applied to relatively short and noisy data; it is largely independent of the length of time series, displays relative consistency under circumstances and has been widely used in physiological studies with different signals analyses (Richman and Moorman, 2000). SampEn (m,r,N) is precisely the negative natural logarithm of the conditional probability that two vectors that are similar for m points remain similar at the next point, where self-matches are not included in calculating the probability (2).


(2) $$\text{SampEn}\,\left(m,r,N\right)=-l\,n\,\frac{A}{B},$$

The parameter N is the length of the time series, m is the length of vectors to be compared, and r is the tolerance for accepting matches. A is the number of pairs of vectors (x) for m points that satisfy the condition d[xm(i), xm(j)] ≤ r, and B is the number of pairs of vectors (x) for (m+1) points that satisfy the condition d[xm(i), xm(j)] ≤ r. Thus, a low value of SampEn reflects a high degree of regularity. SampEn values are largely independent of the time series length and display immunity to signal noise. The length of the analyzed time series was within the range 200–400 RR-intervals. The parameters m and r were fixed: *m* = 2, *r* = 0.2 × SDNN (SDNN—standard deviation of RR-intervals distributions).

Additionally, the time domain indexes of HRV (mean (av-RR, ms) and standard deviation (SDNN, ms) of RR-intervals) were calculated to characterize the average level and general variance of heart rate dynamics.

### Statistical Data Analyses

Statistical analyses were performed using IBM SPSS 17.0 software and open-sourced python SciPy library.^2^ Distributions were tested for normality using Kolmogorov-Smirnov tests. Where distributions were not different from the normal distribution, parametric statistics was applied: *t*-tests for independent samples for between groups comparisons and paired samples *t*-tests for comparisons within subjects. Where distributions differed from the normal distribution, non-parametric tests were used: Mann-Whitney U test, to compare between groups, and Wilcoxon signed-rank and Friedman tests, to compare conditions within subjects. Cohen’s d was calculated as an estimate of effect size for parametric tests. Spearman’s rank correlation coefficient was computed for analyses of relationships between AHS scores, behavioral metrics and HRV indexes. We used an alpha level of 0.05 for all statistical tests.

## Results

### Behavioral Performance in Holistic and Analytic Tasks

#### Response Times

Response times (RTs) were averaged after excluding RT values exceeding two standard deviation units in either direction. RTs for each subject were distributed normally (Kolmogorov-Smirnov test). No difference from the normal distribution was observed for RTs in the entire sample or in separate groups of analytic and holistic participants.

**FIGURE 2:**
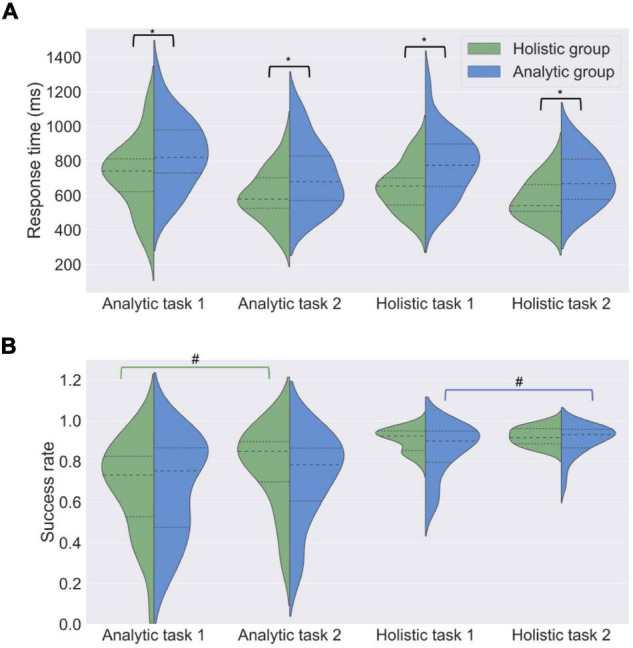
Task performance in analytic and holistic groups. Distribution shapes, median values (dashed lines) and quartiles (dotted lines) are shown for response time (ms) **(A)** and success rate **(B)** in analytic and holistic tasks. Analytic participants solved the cognitive tasks slower than holistic participants. No difference in success rate was observed. *T*-tests for independent samples, *p < 0.05; # - paired samples *t*-tests, ^#^*p* < 0.05.

RTs were compared between the analytic and holistic groups when they were solving tasks in analytic and holistic conditions. The results are presented in Figure 2A (in addition, see Supplementary Table 1). In general, when comparing RTs for the entire sample, analytic tasks (*M* = 778, *SD* = 201) were shown to require longer solution time than holistic tasks (*M* = 718, *SD* = 182) during the first presentation [paired samples *t*-test, *t*(51) = 2.35, *p* = 0.02]; however, solution time did not differ between the two types of tasks (*M* = 667, *SD* = 179 for analytic tasks; *M* = 640, *SD* = 165 for holistic tasks) during the second presentation [paired samples *t*-test, *t*(51) = 1.66, *p* = 0.10].

Holistic participants solved all tasks faster than analytic participants (first block of holistic tasks: *M* = 781, *SD* = 182 for analytic participants, *M* = 651, *SD* = 132 for holistic participants, *t*(45) = 2.78, *p* = 0.01, *d* = 0.83; second block of holistic tasks: *M* = 688, *SD* = 146 for analytic participants, *M* = 570; *SD* = 120 for holistic participants, *t*(45) = 3.00, *p* = 0.01, *d* = 0.44; first block of analytic tasks: *M* = 844, *SD* = 200 for analytic participants, *M* = 719, *SD* = 202 for holistic participants, *t*(45) = 2.11, *p* = 0.05, *d* = 0.37; second block of analytic tasks: *M* = 716, *SD* = 191 for analytic participants, *M* = 601, *SD* = 144 for holistic participants, *t*(45) = 2.31, *p* = 0.05; *d* = 0.34). As can be seen from the effect sizes, this was more pronounced for the holistic tasks.

In addition, tasks were always solved faster during the second presentation, as compared to the first presentation in the whole sample [analytic tasks: *M* = 778, *SD* = 201 for the first presentation, *M* = 667, *SD* = 179 for the second presentation, *t*(51) = 5.29, *p* < 0.001; holistic tasks: *M* = 718, *SD* = 182 for the first presentation, *M* = 640, *SD* = 165 for the second presentation, *t*(51) = 5.27, *p* < 0.001] as well as separately in analytic [analytic tasks: *M* = 844, *SD* = 200 for the first presentation, *M* = 716; *SD* = 191 for the second presentation, *t*(23) = 4.90, *p* < 0.001; holistic tasks: *M* = 781, *SD* = 182 for the first presentation; *M* = 688, *SD* = 146 for the second presentation, *t*(23) = 3.74, *p* < 0.001] and holistic [analytic tasks: *M* = 719, *SD* = 202 for the first presentation, *M* = 601, *SD* = 144 for the second presentation, *t*(22) = 3.55, *p* < 0.001; holistic tasks: *M* = 651, *SD* = 132 for the first presentation, *M* = 570; *SD* = 120 for the second presentation, *t*(22) = 4.17, *p* < 0.001] groups.

Results of correlational analysis (see Table 1) showed reliable negative linear relationships between AHS scores and response times across all tasks, including the simple motor task and cognitive (analytic and holistic) tasks.

**TABLE 1:** Spearman’s rank correlation coefficients for relationships between behavioral metrics (response time (ms), success rate), HRV indexes (av-RR, SDNN and SampEn) at different stages of the experiment and subjects AHS scores (*p < 0.05).

Stage	av_RR	SDNN	SampEn	Response time	Success rate
Rest	−0.13	−0.03	−0.12		
Motor task	−0.13	−0.03	−0.13	−0.30*	
Holistic tasks	−0.14	0.04	−0.26*	−0.31*	0.02
Analytic tasks	−0.12	0.03	−0.21*	−0.29*	−0.05

#### Success Rate

The results are presented in Figure 2B (in addition, see Supplementary Table 2). No difference was found between analytic and holistic groups in the percent of correct solutions [first block of holistic tasks: *M* = 85.4, *SD* = 12.6 for analytic participants, *M* = 90.8, *SD* = 5.5 for holistic participants, *t*(45) = –0.41, *p* = 0.67; first block of analytic tasks: *M* = 66.7, *SD* = 23.9 for analytic participants, *M* = 67.2, *SD* = 23.0 for holistic participants, *t*(45) = –0.06, *p* = 0.94; second block of holistic tasks: *M* = 90.4, *SD* = 7.6 for analytic participants, *M* = 92.4, *SD* = 4.4 for holistic participants, *t*(45) = –1.05, *p* = 0.29; second block of analytic tasks: *M* = 72.0, *SD* = 21.4 for analytic participants, *M* = 77.3, *SD* = 20.0 for holistic participants, *t*(45) = –0.86, *p* = 0.38].

Analysis of task performance within groups showed the following results. Participants were less successful at solving analytic tasks than holistic tasks at the first [*M* = 67.2, *SD* = 22.4 for analytic tasks, *M* = 87.8, *SD* = 9.9 for holistic tasks, *t*(50) = –6.30, *p* < 0.001] and second [*M* = 74.9, *SD* = 19.8 for analytic tasks, *M* = 91.4, *SD* = 6.5 for holistic tasks, *t*(50) = –5.91, *p* < 0.001] presentations. These results were also observed separately in analytic [first presentation: *M* = 66.7, *SD* = 23.9 for analytic tasks, *M* = 85.4, *SD* = 12.6 for holistic tasks, *t*(23) = –3.72, *p* < 0.001; second presentation: *M* = 72.0, *SD* = 21.4 for analytic tasks, *M* = 90.4, *SD* = 7.6 for holistic tasks, *t*(22) = –4.09, *p* < 0.001] and holistic [first presentation: *M* = 67.2, *SD* = 23.0 for analytic tasks, *M* = 90.8, *SD* = 5.5 for holistic tasks, *t*(21) = –4.76, *p* < 0.001; and second presentation: *M* = 77.3, *SD* = 20.0 for analytic tasks, *M* = 92.4, *SD* = 4.4 for holistic tasks, *t*(21) = –3.66, *p* < 0.001] groups.

Success rate of the entire sample significantly increased in the second block of tasks compared with the first block of tasks in both analytic [*M* = 67.2, *SD* = 22.4 for the first presentation, *M* = 74.9, *SD* = 19.8 for the second presentation, *t*(51) = –3.51, *p* < 0.001] and holistic [*M* = 87.8, *SD* = 9.9 for the first presentation, *M* = 91.4, *SD* = 6.5 for the second presentation, *t*(49) = –3.27, *p* < 0.001] conditions, probably reflecting the learning effect. However, in the analytic group, this temporal dynamics was significant only for holistic («nonspecific») tasks [*M* = 85.4, *SD* = 12.6 for the first presentation; *M* = 90.4, *SD* = 7.6 for the second presentation, *t*(22) = –2.42, *p* = 0.02] while no significant shift was observed for analytic tasks [*M* = 66.7, *SD* = 23.9 for the first presentation; *M* = 72.0, *SD* = 21.4 for the second presentation, *t*(23) = –1.73, *p* = 0.09]. Similarly, in the holistic group, significant dynamics in success rate was observed in analytic («nonspecific») tasks [*M* = 67.2, *SD* = 23.0 for the first presentation, *M* = 77.3; *SD* = 20.0 for the second presentation, *t*(22) = –2.74, *p* = 0.01], with only a tendency observed for holistic tasks [*M* = 90.8, *SD* = 5.5 for the first presentation, *M* = 92.4, *SD* = 4.4 for the second presentation, *t*(21) = –1.88, *p* = 0.07].

No correlation was observed between AHS score and success rate (see Table 1).

### Comparing Heart Rate Variability Indexes Between Holistic and Analytic Groups

**FIGURE 3:**
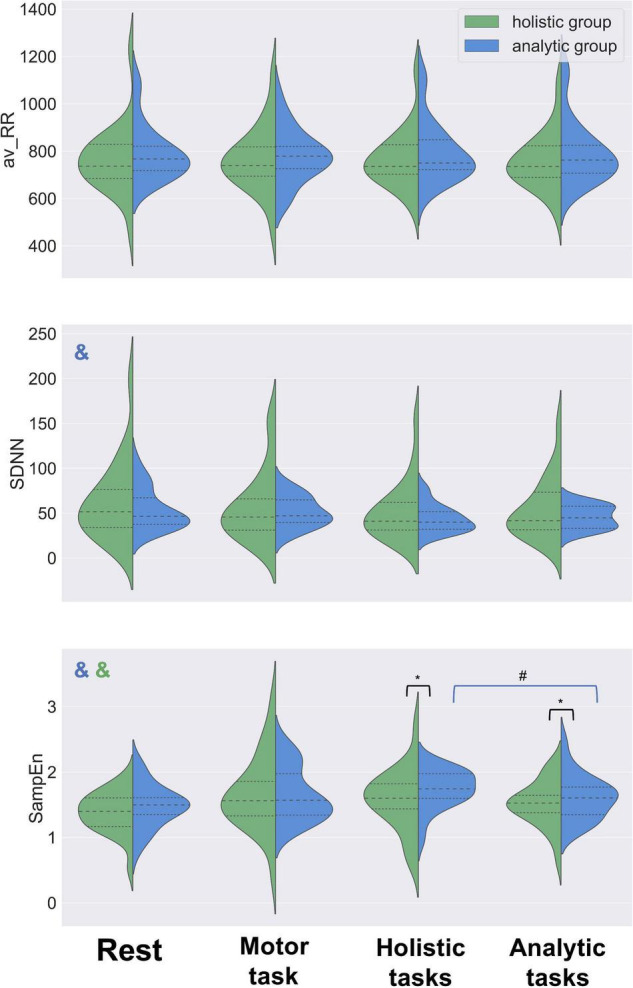
HRV dynamics in analytic and holistic groups. Distribution shapes, median values (dashed lines) and quartiles (dotted lines) are shown for av-RR, SDNN and SampEn estimates. No significant difference between the groups was found in av-RR or SDNN values for baseline, motor task or either type of the cognitive tasks. Higher values of SampEn were observed in the analytic group, as compared with the holistic group, in both analytic and holistic types of tasks. No difference in SampEn was observed between analytic and holistic groups in the baseline or during the motor task. SampEn was increasing from the rest (baseline) condition to the experimental tasks in both groups. SDNN was decreasing from the rest (baseline) condition to the experimental tasks in the analytic group of participants, and their SampEn was higher during solving holistic tasks, as compared to analytic tasks. Mann-Whitney U test, *p < 0.05; Wilcoxon test, ^#^*p* < 0.05; Friedman test, ^&^*p* < 0.05 (color of & reflects the group of analysis).

We compared HRV indexes (SampEn, SDNN, av-RR) between holistic and analytic groups in four conditions: baseline, simple motor task, analytic and holistic blocks of experimental tasks. The results are presented in Figure 3 (in addition, see Supplementary Table 3).

Higher values of SampEn were observed in analytic participants, as compared with holistic participants, in both analytic (Mann-Whitney test, *U* = 186, *p* = 0.04) and holistic (Mann-Whitney test, *U* = 177, *p* = 0.02) tasks. No difference in SampEn was observed between analytic and holistic groups in the baseline condition (Mann-Whitney test, *U* = 196, *p* = 0.20) or during the simple motor task (Mann-Whitney test, *U* = 240, *p* = 0.38). No significant difference between the groups was found in SDNN values for any of the four conditions: baseline (Mann-Whitney test, *U* = 224, *p* = 0.43), simple motor task (Mann-Whitney test, *U* = 243, *p* = 0.41), analytic tasks (Mann-Whitney test, *U* = 255, *p* = 0.42) or holistic tasks (Mann-Whitney test, *U* = 243, *p* = 0.32). No difference between the groups was observed in the length of RR-intervals (av-RR) for baseline (Mann-Whitney test, *U* = 200, *p* = 0.23), simple motor task (Mann-Whitney test, *U* = 239, *p* = 0.38), analytic tasks (Mann-Whitney test, *U* = 215, *p* = 0.14) or holistic tasks (Mann-Whitney test, *U* = 221, *p* = 0.17).

In addition, we further compared the HRV indexes between the subgroups of participants with the highest analytic scores (AHS score below 75% of the threshold of the sample distribution, *N* = 18) and highest holistic scores (AHS score above 25% of the threshold of the sample distribution, *N* = 17). Despite the smaller sample sizes, this contrast replicated the findings shown for the entire sample. Higher values of SampEn were observed in participants with high analytic scores, as compared with participants with high holistic scores, in both types of tasks: analytic (Mann-Whitney test, *U* = 95, *p* = 0.02) and holistic (Mann-Whitney test, *U* = 98, *p* = 0.03). SampEn did not differ between the subgroups in the baseline condition (Mann-Whitney test, *U* = 117, *p* = 0.35) or during the simple motor task (Mann-Whitney test, *U* = 115, *p* = 0.16). No difference between the subgroups was observed in SDNN values for the baseline (Mann-Whitney test, *U* = 124, *p* = 0.45), simple motor task (Mann-Whitney test, *U* = 138, *p* = 0.42), analytic tasks (Mann-Whitney test, *U* = 143, *p* = 0.37), or holistic tasks (Mann-Whitney test, *U* = 151, *p* = 0.48). No difference was found in av-RR values: baseline (Mann-Whitney test, *U* = 117, *p* = 0.35), simple motor task (Mann-Whitney test, *U* = 142, *p* = 0.47), analytic tasks (Mann-Whitney test, *U* = 126, *p* = 0.19), and holistic tasks (Mann-Whitney test, *U* = 126, *p* = 0.19).

Results of correlational analysis (see Table 1) showed reliable negative linear relationships between AHS score and SampEn values in analytic and holistic tasks. No correlation was found between AHS score and other HRV indexes (SDNN and av-RR).

### Dynamics of Heart Rate Variability Indexes Within Analytic and Holistic Groups During the Experiment

We studied the dynamics of HRV indexes in analytic and holistic groups during four experimental conditions: rest, simple motor task, holistic and analytic tasks. HRV indexes were compared across the above conditions using Friedman test followed by pairwise comparisons using Wilcoxon test. These results are presented in Figure 3.

#### Dynamics of Heart Rate Variability Indexes in the Analytic Group

Significant difference in SampEn values was shown between analytic and holistic tasks (Wilcoxon test, *T* = 72, *p* = 0.04). In general, SampEn was increasing from the rest condition to experimental tasks (Friedman test, *Q* = 11.35, *p* = 0.01), with the highest values observed during the holistic tasks.

SDNN dynamics was also significant: the index values decreased from the rest condition to experimental tasks (Friedman test, *Q* = 10.42, *p* = 0.02), which may be accounted for by light fatigue participants developed during the experiment. However, no difference was observed between the analytic and holistic tasks (Wilcoxon test, *T* = 95, *p* = 0.19).

No trend was found for av-RR (Friedman test, *Q* = 0.71, *p* = 0.87) and no difference in av-RR was shown between the analytic and holistic tasks (Wilcoxon test, *T* = 136, *p* = 0.95).

#### Dynamics of Heart Rate Variability Indexes in the Holistic Group

SampEn was increasing from the rest condition to the experimental tasks in the holistic group (Friedman test, *Q* = 8.54, *p* = 0.04). No difference was found between the analytic and holistic tasks (Wilcoxon test, *T* = 126, *p* = 0.72).

SDNN dynamics was not significant between the experimental conditions (Friedman test, *Q* = 0.97, *p* = 0.58); and no difference was observed between the analytic and holistic tasks (Wilcoxon test, *T* = 112, *p* = 0.42).

No difference was shown for av-RR dynamics between the experimental conditions (Friedman test, *Q* = 0.49, *p* = 0.92) or between the analytic and holistic tasks (Wilcoxon test, *T* = 96, *p* = 0.20).

## Discussion

In this study, we tested the hypothesis that cognitive and behavioral specificity of analytic and holistic thinking styles is manifested in the dynamics of neuro-visceral processes, reflecting the system organization of individual experience. Our results have shown differences in behavioral performance and heart rate complexity between analytic and holistic thinkers when solving cognitive tasks designed to contrast analytic and holistic conditions, i.e., instructing to focus on either an object itself or the same object in relation to the field it is presented with. More specifically, longer response times and higher heart rate complexity values were observed in the analytic group compared with the holistic group, with no difference in the number of correct solutions between the groups.

As mentioned above, analytic participants tended to solve all tasks, in both holistic and analytic conditions, slower than holistic participants; and this difference in response times was more prominent for holistic tasks. Both groups were equally successful at solving the tasks and delivered similar numbers of correct responses. These results on task performance reflect the fact that holistic and analytic individuals use different behavioral and problem-solving strategies, which had been described previously (Norenzayan et al., 2002; Choi et al., 2007). As mentioned in the introduction, one of the key features of holistic thinking is a propensity to evaluate events and objects in relation to the context and pay attention to various links between them and the environment. In contrast, analytic individuals tend to consider events and objects as separate and invariant in time, primarily changing according to their own rules, rather than due to interaction with the environment (Nisbett, 2003). In our tasks, holistic participants may have relied on the relationship between the objects (lines) and their fields (frames) which helped them to make comparative judgments faster in both conditions, when the field was part of the instruction and when it was not. In contrast, analytic thinkers used pre-set criteria to select individual objects in the environment and evaluate them independently. In other words, while holistic thinkers tended to classify objects on the basis of their general relationship to the field, analytic thinkers were inclined to rely on categorization rules and formal logic, evaluating given objects separate from their context. Due to no difference in success rate but slower response times in analytic participants, it may be suggested that the tasks used in our study were harder for them to solve, and more so in the holistic condition.

In line with the behavioral performance, we have shown that analytic individuals had significantly higher heart rate complexity, as measured by SampEn, than holistic individuals while solving both analytic and holistic experimental tasks. This difference between the groups was related to the temporal dynamics observed in analytic individuals, whose heart rate complexity significantly increased from the rest condition toward the experimental tasks and was higher during the tasks with holistic conditions compared to the tasks with analytic conditions. In the group of holistic individuals, heart rate complexity showed similar dynamics, but no difference was observed between the tasks with holistic and analytic conditions. These results on differences in heart rate complexity are consistent with our previous EEG data recorded while individuals were solving the same experimental tasks: we had shown that analytic thinkers had higher variability of P300 component of ERP than holistic thinkers (Apanovich et al., 2021). In another study, fMRI was performed while individuals were watching a film depicting socioemotional interactions: it was shown that holistic thinkers had significant inter-subject correlations in more extensive cortical areas than analytic thinkers, suggesting that they perceived the content of the film in a more similar fashion (Bacha-Trams et al., 2018). In addition, the authors found that participants’ eye gaze patterns were more uniform/correlated in the group of holistic thinkers compared to analytic thinkers; and higher variation in breathing rate was observed in the analytic group while no difference was detected between the two groups in averaged heart rate frequency. These results and our findings indicate that individuals with holistic thinking style tend to display less variation in behavioral and psychophysiological measures, both in the brain and in visceral activity, while engaging in problem-solving and other activities.

It is important to note that neither our work, nor the study by Bacha-Trams et al. (2018) mentioned above, found differences in averaged inter-beat intervals. This measure reflects more general and intensive changes in physiological energy levels. Standard deviation of RR-intervals (SDNN) decreased in the group of analytic participants throughout the experiment. Such SDNN dynamics in combination with constant averaged heart rate is usually related to a decrease in individual energy levels due to experiencing light fatigue (Boneva et al., 2007; Iizuka et al., 2020). This is also supported by the fact that analytic participants solved the tasks slower, suggesting higher cognitive effort. No difference between analytic and holistic groups in SDNN values indicate a similar general level of the autonomic nervous system activity. SDNN and SampEn metrics reflect different aspects of HRV and differences in their dynamics can be observed for certain modes of activity: for example, a decrease in general variation of the time sequence of RR-intervals can simultaneously be accompanied by an increase in non-stationarity of their internal structure. Therefore, we suggest that the difference in heart rate complexity observed between analytic and holistic thinkers is related to higher levels of neuro-visceral integration, i.e., activity of neuronal groups in the cortex involved in regulation of the heart rate related to behavior (Smith et al., 2017; Bakhchina et al., 2018).

Cognitive tasks, especially attention tests, are often used in the studies of neuronal structures involved in the regulation of heart rate (Napadow et al., 2008; Lane et al., 2009). As a result, the current models of neuro-visceral coordination demonstrate correlations between brain activity in various cortical and subcortical regions with HRV dynamics and cognitive processes (Smith et al., 2017). In particular, positive correlations are shown between neuronal activity in the cingulate cortex, cognitive performance and nonlinear characteristics of HRV along with directly proportional relationships between cingulate cortex morphology (thickness, cell density) and characteristics of HRV at rest (Winkelmann et al., 2017). Thus, the difference in heart rate complexity observed between analytic and holistic thinkers can also be considered in the framework of organization of physiological activity in the brain and the rest of the body as a foundation of behavioral performance.

As described in the Introduction, one of the key differences between analytic and holistic thinkers is their attentional focus on either separate objects or contextual relationships between objects. For example, holistic strategies are often characterized as involving the global precedence effect, i.e., when the global-level properties are prioritized in cognitive processing compared to the local properties (Wagemans et al., 2012). The difference in heart rate complexity between analytic and holistic thinkers observed in our study can be interpreted in terms of using different cognitive strategies. At the same time analytic vs. holistic thinking is a wider concept which is used as a psychological meta-category (Masuda and Nisbett, 2001; Kitayama et al., 2003) describing two different ways of organization of individual experience (Apanovich et al., 2018). Thus, analytic vs. holistic thinking includes many local attentional and perceptual specificities that, being considered separately, also correlate with non-linear HRV metrics (Forte et al., 2019). As an illustrative example, it had been shown previously in the studies contrasting individuals with different political views that, on average, conservatives are more likely to use holistic types of classification based on relationships between objects, whereas liberals more frequently use analytical taxonomic classification of objects (Amodio et al., 2007; Talhelm et al., 2015); at the same time greater liberalism is associated with higher gray matter volume in the anterior cingulate cortex, whereas greater conservatism is associated with higher volume in the right amygdala (Kanai et al., 2011). Cingulate cortex and amygdala are shown to be the two key sources of HRV across cortical structures due to their morphological and functional relations with the main centers of the autonomic nerve system in the brainstem (Smith et al., 2017). Therefore, even such aspect of cognitive activity as political judgment can be reflected in HRV. In line with the above, autonomic changes representing sympathetic activity and measured by skin conductance during viewing of threatening images were shown to be more pronounced in conservatives (Oxley et al., 2008).

From the perspective of the system-evolutionary theory, HRV originates in cooperation of the heart with the other components of actualized functional systems, including neuronal groups, activity of which underlies behavior at the level of the whole organism. In this view, non-linear changes in heart rate dynamics reflect coordination of heart activity with changes in the sets of activated neurons distributed across the brain and supporting current behavior. From this perspective, the results of our study may be used to speculate about possible differences in the structure of individual experience between analytic and holistic thinkers. While solving analytic and holistic tasks during the experiment, our participants were learning new behavior. In the holistic group, heart rate complexity was lower and more constant because the task was easier for them to solve, which was reflected in faster response times, and their learning processes involved more recombination of existing elements of experience rather than creating new elements (e.g., see Aleksandrov, 2006; Kuzina and Alexandrov, 2017). In analytic thinkers, the learning process involved a more extended creation of new elements and their addition into the structure of individual experience, which required more resources and their regulation. Thus, higher heart rate complexity and longer response times, especially in holistic tasks, reflect more intense learning processes in analytic individuals.

In all comparisons of response times, the tasks were solved faster at the second presentation compared to the first presentation, which points to the general effect of learning. Interestingly, along with faster responses during the second presentation, success rate increased selectively only in holistic tasks for analytic participants and only in analytic tasks for holistic participants. This task specific dynamics of success rate suggests that during our experiment holistic thinkers were learning to better solve analytic tasks and analytic thinkers were improving at solving holistic tasks. At the same time, the increase in heart rate complexity was less pronounced in the holistic group and its values during experimental tasks were closer to the rest condition, as compared with the analytic group. This is consistent with our assumptions that different system mechanisms may underlie seemingly similar learning dynamics, and that differences in the structure of experience are manifested in heart rate complexity.

## Conclusion

Overall, our findings demonstrate that differences in individuals’ behavior, including those related to holistic and analytic thinking styles, can be reflected not only in brain activity, as it had been shown previously in other studies using fMRI and EEG, but can also be observed at the level of neuro-visceral activity as manifested in heart rate complexity. Our results support the hypothesis about differences in neuro-visceral coordination supporting behavior while solving cognitive tasks between analytic and holistic individuals and that it may vary across tasks and conditions. The holistic (systemic) approach to achieving a deeper understanding of analytic and holistic thinking requires further investigation into the nature of psychophysiological organization of behavior in analytic and holistic thinkers.

## Data Availability Statement

The original contributions presented in the study are included in the article/Supplementary Material, further inquiries can be directed to the corresponding author/s.

## Ethics Statement

The studies involving human participants were reviewed and approved by the Ethics Committee of Institute of Psychology of Russian Academy of Sciences (Moscow). The patients/participants provided their written informed consent to participate in this study.

## Author Contributions

AB performed the experiments, analyzed, interpreted the data, contributed reagents, materials, analysis tools or data, and wrote the manuscript. VA performed the experiments, analyzed, and interpreted the data. KA conceived, designed the experiments, performed the experiments, analyzed, interpreted the data, and wrote the manuscript. YA conceived, designed the experiments, analyzed, and interpreted the data. All authors contributed to the article and approved the submitted version.

## Conflict of Interest

The authors declare that the research was conducted in the absence of any commercial or financial relationships that could be construed as a potential conflict of interest.

## Publisher’s Note

All claims expressed in this article are solely those of the authors and do not necessarily represent those of their affiliated organizations, or those of the publisher, the editors and the reviewers. Any product that may be evaluated in this article, or claim that may be made by its manufacturer, is not guaranteed or endorsed by the publisher.

## Footnotes

1
http://medicom-mtd.com/

2
https://www.scipy.org/

## References

[B1] Acharya U. R. Joseph K. P. Kannathal N. Lim C. M. Suri J. S. (2006). Heart rate variability: a review. *Med. Biol. Eng.* 44 1031–1051. 10.1007/s11517-006-0119-0 17111118

[B2] Aleksandrov Y. I. (2006). Learning and memory: traditional and systems approaches. *Neurosci. Behav. Physiol.* 36 969–985. 17024336 10.1007/s11055-006-0133-6

[B3] Amodio D. M. Jost J. T. Master S. L. Yee C. M. (2007). Neurocognitive correlates of liberalism and conservatism. *Nat. Neurosci.* 10 1246–1247. 10.1038/nn1979 17828253

[B4] Anokhin P. K. (1974). *Biology and Neurophysiology of Conditioned Reflex and Its Role in Adaptive Behavior.* Oxford: Pergamon Press.

[B5] Apanovich V. V. Aramyan E. A. Dol’nikova M. S. Aleksandrov Y. I. (2021). Differences in brain support for solving analytical and holistic problems. *Psikholog. Zh.* 42 45–60. 10.31857/S020595920014240-0

[B6] Apanovich V. V. Bezdenezhnykh B. N. Sams M. Jääskeläinen I. P. Alexandrov Y. I. (2018). Event-related potentials during individual, cooperative, and competitive task performance differ in subjects with analytic vs. holistic thinking. *Int. J. Psychophysiol.* 123 136–142. 10.1016/j.ijpsycho.2017.10.001 28986326

[B7] Apanovich V. V. Bezdenezhnykh B. N. Znakov V. V. Sams M. Jaaskelainen J. Aleksandrov Y. I. (2016). Differences of the brain activity in individual, competitive and cooperative behavior between subjects with analytic and holistic cognitive styles. *Eksperimental’naya psikhologiya* 9 5–22.

[B8] Apanovich V. V. Tishchenko A. G. Znakov V. V. Aleksandrov Y. I. (2020). Construction of blocks of analytical and holistic problems and their empirical verification. *Vopr. Psikhol.* 66 142–154.

[B9] Apanovich V. V. Znakov V. V. Aleksandrov Y. I. (2017). Approbation of the russian-language version of Analytic-Holistic scale. *Psikholog. Zh.* 28 80–96. 10.7868/S0205959217050075

[B10] Arutyunova K. R. Bakhchina A. V. Sozinova I. M. Alexandrov Y. I. (2020). Complexity of heart rate variability during moral judgement of actions and omissions. *Heliyon* 16:e05394. 10.1016/j.heliyon.2020.e05394 33235931 PMC7672222

[B11] Bacha-Trams M. Alexandrov Y. I. Broman E. Glerean E. Kauppila M. Kauttonen J. (2018). A drama movie activates brains of holistic and analytical thinkers differentially. *Soc. Cogn. Affect. Neurosci.* 13 1293–1304. 10.1093/scan/nsy099 30418656 PMC6277741

[B12] Bakhchina A. V. Arutyunova K. R. Sozinov A. A. Demidovsky A. V. Alexandrov Y. I. (2018). Sample entropy of the heart rate reflects properties of the system organization of behaviour. *Entropy* 20:449. 10.3390/e20060449 33265539 PMC7512967

[B13] Baranski J. V. Petrusic W. M. (1999). Realism of confidence in sensory discrimination. *Percept. Psychophys.* 61 1369–1383. 10.3758/bf03206187 10572465

[B14] Boneva R. S. Decker M. J. Maloney E. M. Lin J. M. Jones J. F. Helgason H. G. (2007). Higher heart rate and reduced heart rate variability persist during sleep in chronic fatigue syndrome: a population-based study. *Auton. Neurosci.* 137 94–101. 10.1016/j.autneu.2007.08.002 17851136

[B15] Choi I. Koo M. Choi J. A. (2007). Individual differences in analytic versus holistic thinking. *Pers. Soc. Psychol. Bull.* 33:691. 10.1177/0146167206298568 17440200

[B16] Choi I. Nisbett R. E. Smith E. E. (1997). Culture, categorization and inductive reasoning. *Cognition* 65 15–32. 9455169 10.1016/s0010-0277(97)00034-6

[B17] Forte G. Favieri F. Casagrande M. (2019). Heart rate variability and cognitive function: a systematic review. *Front. Neurosci.* 13:710. 10.3389/fnins.2019.00710 31354419 PMC6637318

[B18] Haun D. B. Rapold C. J. Call J. Ganzen G. Levinson S. C. (2006). Cognitive cladistics and cultural override in hominid spatial cognition. *Proc. Natl. Acad. Sci. U.S.A.* 103 17568–17573. 10.1073/pnas.0607999103 17079489 PMC1859970

[B19] Hedden T. Ketay S. Aron A. Markus H. R. Gabrieli J. (2008). Cultural influences on neural substrates of attentional control. *Psychol. Sci.* 19 12–17. 10.1111/j.1467-9280.2008.02038.x 18181784

[B20] Hilgarter K. Schmid-Zalaudek K. Csanády-Leitner R. Mörtl M. Rössler A. Lackner H. K. (2021). Phasic heart rate variability and the association with cognitive performance: a cross-sectional study in a healthy population setting. *PLoS One* 16:e0246968. 10.1371/journal.pone.0246968 33647023 PMC7920382

[B21] Hsieh S. Yu Y.-T. Chen E.-H. Yang C.-T. Wang C.-H. (2020). ERP correlates of a flanker task with varying levels of analytic-holistic cognitive style. *Pers. Individ. Dif.* 153:109673. 10.1016/j.paid.2019.109673

[B22] Huang C. M. Doole R. Wu C. W. Huang H. W. Chao Y. P. (2019). Culture-related and individual differences in regional brain volumes: a cross-cultural voxel-based morphometry study. *Front. Hum. Neurosci.* 13:313. 10.3389/fnhum.2019.00313 31551740 PMC6746838

[B23] Iizuka T. Ohiwa N. Atomi T. Shimizu M. Atomi Y. (2020). Morning heart rate variability as an indication of fatigue status in badminton players during a training camp. *Sports* 8:147. 10.3390/sports8110147 33182645 PMC7697084

[B24] Ji L. J. Nisbett R. Su Y. (2001). Culture, change, and prediction. *Psychol. Sci.* 12 450–456. 10.1111/1467-9280.00384 11760130

[B25] Ji L. J. Peng K. Nisbett R. E. (2000). Culture, control, and perception of relationships in the environment. *J. Pers. Soc. Psychol.* 78 943–955. 10.1037//0022-3514.78.5.943 10821200

[B26] Kanai R. Feilden T. Firth C. Rees G. (2011). Political orientations are correlated with brain structure in young adults. *Curr. Biol.* 21 677–680. 10.1016/j.cub.2011.03.017 21474316 PMC3092984

[B27] Kemp A. H. Quintanaa D. S. Grayd M. A. Felminghamb K. L. Brownc K. Gattc J. M. (2010). Impact of depression and antidepressant treatment on heart rate variability: a review and meta analysis. *Biol. Psychiatry* 67 1067–1074. 10.1016/j.biopsych.2009.12.012 20138254

[B28] Kitayama S. Duffy S. Kawamura T. Larsen J. T. (2003). Perceiving an object and it’s context in different cultures: a cultural look at new look. *Psychol. Sci.* 14 201–206. 10.1111/1467-9280.02432 12741741

[B29] Kuzina E. A. Alexandrov Y. I. (2017). “Everyday repetition of the instrumental behavior and reorganization of the associated brain activity,” in *Fundamental and Applied Research in Modern Psychology: Results and Development Prospects*, eds Zhuravlev A. L. Koltsova V. A. (Moscow: Institute of Psychology RAS), 1583–1591.

[B30] Laborde S. Mosley E. Thayer J. F. (2017). Heart rate variability and cardiac vagal tone in psychophysiological research – recommendations for experiment planning, data analysis, and data reporting. *Front. Psychol.* 8:213. 10.3389/fpsyg.2017.00213 28265249 PMC5316555

[B31] Lane R. D. McRae K. Reiman E. M. Chen K. Ahern G. L. Thayer J. F. (2009). Neural correlates of heart rate variability during emotion. *Neuroimage* 44 213–222. 10.1016/j.neuroimage.2008.07.056 18778779

[B32] Macedonia M. (2019). Embodied learning: why at school the mind needs the body. *Front. Psychol.* 10:2098. 10.3389/fpsyg.2019.02098 31632311 PMC6779792

[B33] Makowski D. Pham T. Lau Z. J. Brammer J. C. Lespinasse F. Pham H. (2021). NeuroKit2: a python toolbox for neurophysiological signal processing. *Behav. Res. Methods* 53 1689–1696. 10.3758/s13428-020-01516-y 33528817

[B34] Masuda T. Nisbett R. E. (2001). Attending holistically versus analytically: comparing the context sensitivity of Japanese and Americans. *J. Pers. Soc. Psychol.* 81:922. 10.1037//0022-3514.81.5.922 11708567

[B35] Napadow V. Dhond R. Conti G. Makris N. Brown E. N. Barbieri R. (2008). Brain correlates of autonomic modulation: combining heart rate variability with Fmri. *Neuroimage* 42 169–177. 10.1016/j.neuroimage.2008.04.238 18524629 PMC2603289

[B36] Nisbett R. E. (2003). *The Geography of Thought: How Asians and Westerners Think Differently, and Why.* New York, NY: Free Press.

[B37] Nisbett R. E. Peng K. Choi I. Norenzayan A. (2001). Culture and systems of thought: holistic versus analytic cognition. *Psychol. Rev.* 108 291–310. 10.1037/0033-295X.108.2.291 11381831

[B38] Norenzayan A. Smith E. Kim B. Nisbett R. (2002). Cultural preferences for formal versus intuitive reasoning. *Cogn. Sci.* 26 653–684. 10.1016/S0364-0213(02)00082-4

[B39] Oxley D. R. Smith K. B. Alford J. R. Hibbing M. V. Miller J. L. Scalora M. (2008). Political attitudes vary with physiological traits. *Science* 321 1667–1670. 10.1126/science.1157627 18801995

[B40] Pan J. Tompkins W. J. (1985). A real-time QRS detection algorithm. *IEEE Trans. Biomed. Eng.* 32 230–236. 10.1109/TBME.1985.325532 3997178

[B41] Peng K. Nisbett R. E. (1999). Culture, dialectics, and reasoning about contradiction. *Am. Psychol.* 54 741–754. 10.1037/0003-066X.54.9.741

[B42] Pham T. Lau Z. J. Chen C. H. Makowski D. (2021). Heart rate variability in psychology: a review of HRV indices and an analysis tutorial. *Sensors* 21:3998. 10.3390/s21123998 34207927 PMC8230044

[B43] Richman J. S. Moorman J. R. (2000). Physiological time-series analysis using approximate entropy and sample entropy. *Am. J. Physiol. Heart Circ. Physiol.* 278 2039–2049. 10.1152/ajpheart.2000.278.6.H2039 10843903

[B44] Shvyrkov V. B. (1990). “Neurophysiological study of animals’ subjective experience,” in *Machinery of the Mind*, eds John R. Harmony T. (Boston, MA: Birkhäuser), 337–350.

[B45] Smith R. Thayer J. F. Khalsa S. S. Lane R. D. (2017). The hierarchical basis of neurovisceral integration. *Neurosci. Biobehav. Rev.* 75 274–296. 10.1016/j.neubiorev.2017.02.003 28188890

[B46] Talhelm T. Haidt J. Oishi S. Zhang X. Miao F. F. Chen S. (2015). Liberals think more analytically (more “WEIRD”) than conservatives. *Pers. Soc. Psychol. Bull.* 41 250–267. 10.1177/0146167214563672 25540328

[B47] Tan L. H. Chan A. H. Kay P. Khong P.-L. Yip L. K. Luke K.-K. (2008). Language affects pat- terns of brain activation associated with perceptual decision. *Proc. Natl. Acad. Sci. U.S.A.* 105 4004–4009. 10.1073/pnas.0800055105 18316728 PMC2268832

[B48] Thayer J. F. Lane R. D. (2009). Claude Bernard and the heart-brain connection: further elaboration of a model of neurovisceral integration. *Neurosci. Biobehav. Rev.* 33, 81–88. 10.1016/j.neubiorev.2008.08.004 18771686

[B49] Wagemans J. Elder J. H. Kubovy M. Palmer S. E. Peterson M. A. Singh M. (2012). A century of Gestalt psychology in visual perception: I. perceptual grouping and figure–ground organization. *Psychol. Bull.* 138 1218–1252. 10.1037/a0029334 22845751 PMC3482144

[B50] Winawer J. Witthoft N. Frank M. C. Wu L. Wade A. R. Boroditsky L. (2007). Russian blues reveal effects of language on color discrimination. *Proc. Natl. Acad. Sci. U.S.A.* 104 7780–7785. 10.1073/pnas.0701644104 17470790 PMC1876524

[B51] Winkelmann T. Thayer J. F. Pohlack S. Nees F. Grimm O. Flor H. (2017). Structural brain correlates of heart rate variability in a healthy young adult population. *Brain Struct. Funct.* 222 1061–1068. 10.1007/s00429-016-1185-1 26801184

