# Supplementary material for: Analytic and Holistic Thinkers: Differences in the Dynamics of Heart Rate Complexity When Solving a Cognitive Task in Field-Dependent and Field-Independent Conditions
Source: Front Psychol. 2021 Nov 26;12:762225. doi: 10.3389/fpsyg.2021.762225 (PMC8661497; doi:10.3389/fpsyg.2021.762225)
Supplement: Supplementary file 1 [file Data_Sheet_1.PDF]

Read me

Table 1:

Column name	Description
id_participant	Identical number of the participant
Response time (ms)	Response time in performance of analytic and holistic tasks
group	Group of the participant according to Analysis-holism scale score
task	holistic tasks (1 and 2 experimental sessions (5 min per session) in the holistic condition),<br>analytic tasks (1 and 2 experimental sessions (5 min per session) in the analytic condition).

Table 2:

Column name	Description
id_participant	Identical number of the participant
Success rate	the number of correct solutions in performance of analytic and holistic tasks
group	Group of the participant according to Analysis-holism scale score
task	holistic tasks (1 and 2 experimental sessions (5 min per session) in the holistic condition),<br>analytic tasks (1 and 2 experimental sessions (5 min per session) in the analytic condition).

Table 3:

Column name	Description
id_participant	Identical number of the participant
stage	rest (5 min, while sitting down with closed eyes without moving),<br>motor task (2 min, during a simple motor task involving familiarizing with keys to be used in the following experimental sessions),<br>holistic tasks (average value in the process of two experimental sessions (5 min per session) in the holistic condition),<br>analytic tasks (average value in the process of two experimental sessions (5 min per session) in the analytic condition).
group	Group of the participant according to Analysis-holism scale score
av_RR (ms)	average value of RR-intervals
SDNN (ms)	standard deviation of RR-intervals

SampEn	sample entropy of RR-intervals sequence
